# Association of regenerating gene 1A single-nucleotide polymorphisms and nasopharyngeal carcinoma susceptibility in southern Chinese population

**DOI:** 10.1007/s00405-019-05645-9

**Published:** 2019-09-20

**Authors:** Haijie Xing, Xiangdong Chen, Hongxia Sun, Yaofeng Han, Lanshu Ding, Xiaoxia Chen

**Affiliations:** 1 Department of Otorhinolaryngology, Head and Neck Surgery, Chinese Science Academy University, Shenzhen Hospital, No. 4253 Songbai Road, Shenzhen, 518106 People’s Republic of China; 2 Department of Otorhinolaryngology Head and Neck Surgery, Affiliated Xinhua Hospital, Hainan Medical College, No. 48 Baishuitang Road, Haikou, 570311 People’s Republic of China; 3 Department of Otolaryngology Head and Neck Surgery, Affiliated General Hospital of Shenzhen University, No. 1048 Xueyuan Road, Shenzhen, 518106 People’s Republic of China; 4 Wuhan Medical Science Research Institute, No. 47, Lihuangpi Road, Wuhan, 430013 People’s Republic of China; 5 Department of Epidemiology, Public Health College of Xiamen University, No. 344 Pengxiang Road, Xiamen, 361005 People’s Republic of China; 6 Nursing Department, Chinese Science Academy University, Shenzhen Hospital, No.4253 Songbai Road, Shenzhen, 518106 People’s Republic of China

**Keywords:** Nasopharyngeal, Carcinoma, Regenerating gene 1A, Polymorphism, Susceptibility

## Abstract

**Objective:**

Nasopharyngeal carcinoma (NPC) is a common malignancy in Southern China and Southeast Asia. Genetic susceptibility is a major contributing factor in determining the individual risk of NPC in these areas. To test the association between NPC and variants in *regenerating gene 1A (REG1A)*, we conducted a hospital-based case–control study in a Cantonese-speaking population from Guangdong province.

**Methods:**

We endeavored to determine whether genetic variants of the *REG1A* gene were associated with the risk of NPC amidst the Cantonese population in a hospital-based case–control study using polymerase chain reaction-restriction and direct sequencing analysis in 211 NPC patients and 150 healthy controls. The association between NPC risk and the 14C/T, 20C/T, 369G/T, 1201A/G, and 2922C/T polymorphisms was examined after adjustment for age and sex.

**Results:**

We found an increased risk of developing NPC in individuals with *REG1A* 2922C/T variant genotype (*p* = 0.003, OR 0.419, 95% CI 0.235–0.746), and after adjustment for sex and age (*p* = 0.003, OR 0.406, 95% CI 0.226–0.732). No association between other polymorphisms (14C/T, 20C/T, 369G/T, and 1201A/G) and the risk of NPC was observed, before or after adjustment for age and sex.

**Conclusion:**

Our findings suggest that the *REG1A* 2922C/T polymorphism is associated with an increased risk of developing NPC in a Cantonese population from Guangdong province. Larger studies are required to confirm our findings and unravel the underlying mechanisms.

**Electronic supplementary material:**

The online version of this article (10.1007/s00405-019-05645-9) contains supplementary material, which is available to authorized users.

## Introduction

Nasopharyngeal carcinoma (NPC) originates from the epithelial lining of the nasopharynx [1]. It is characterized by a higher incidence in Southern China and Southeast Asia, at 20–30 per 100,000 people [2]. The etiology of NPC is not clear, but genetic susceptibility, Epstein–Barr virus (EBV) infection and environmental factors are predisposing factors for NPC [3, 4]. Different individuals exposed to the same environmental factors will have distinct presentation, suggesting that genetic variation is the key risk factor for the development of NPC [5].

Many genetic studies have been conducted to address susceptibility genes for NPC and, using linkage approach or association analyses, some genes have been identified. −842G>C and −667C>T in PIN1 promoter variants [6], Filaggrin gene (*FLG*) single-nucleotide polymorphism (SNP) loci (rs3126085, K4671X) [7], Cyclin D1 (*CCND1*) [8], and the DNA repair genes *hOGG1* and *XRCC1* [9] seem to contribute to NPC. Some studies have indicated that SNPs are associated with treatment outcomes and prognosis in NPC. SNPs such as rs2074549 in valosin-containing protein, rs7566 in CANX, rs2528521 in CALCR and rs9344 in *CCND1* may serve as predictors for clinical outcomes of chemoradiotherapy in Chinese NPC patients [10, 11]. SNPs like rs1800541, rs2071942 and rs5370 in *EDN1*, rs5333 in *EDNRA* and *VEGF* −460T/C may serve as useful biomarkers for predicting the outcomes of NPC patients [12, 13]. Another recent study has shown that genetic polymorphisms in *MCP-1* and *HLA-G* are associated with NPC prognosis [14, 15]. Despite these advances, the alleles that account for most of the genetic susceptibility to NPC remain undiscovered.

The regenerating gene (*REG*) was originally isolated from a complementary DNA (cDNA) library derived from regenerating rat’s pancreatic islets and consists of a group of acute phase reactants, lectins, anti-apoptotic factors, or growth factors for pancreatic β-islet cells and epithelial cells in the digestive system. Until now, 17 members of the REG family have been identified and classified into four classes (Reg I–IV) [16]. *REG1A* is one of the members of the human *REG1* family, and has six exons encoding a 166-amino-acid protein and a 22-amino-acid signal sequence [17]. In humans, *REG1A* and the other members of the *REG1* family (*REG1B*, RS (REG-related sequence), and *PAP* genes) are clustered in a 95-kb region on chromosome 2p12.

*REG1A* is involved not only in inflammatory diseases, but also in gastroenterological carcinogenesis, including the stomach [18], colon [19], and pancreas [20]. Meanwhile, evidence has revealed that *REG1A* is also related to breast cancer [21], lung cancer [22], recurrence of bladder cancer [23], hepatocellular carcinoma metastasis [24], and prognosis of head and neck tumors [25]. Some recent studies also demonstrated that *REG1A* expression was associated with chemoradiotherapy outcome in patients with esophageal squamous cell cancer [26]. A previous study by our group showed that *REG1A* overexpression was associated with NPC progression and prognosis [27]. Studies had seldom been conducted concerning the *REG1A* polymorphisms in relation to cancer.

This has provided a molecular basis for determining possible associations of *REG1A* polymorphisms and NPC predisposition. In the present case–control study, we first sequenced all six exons of the *REG1A* gene and evaluated for the potential link between the *REG1A* polymorphisms and the risk of NPC among Guangdong Cantonese. We found that the *REG1A* 2922C/T variants are associated with an increased risk of NPC and may be a novel biomarker for the screening and early diagnosis of NPC.

## Materials and methods

### Subjects

A total of 211 patients with NPC were enrolled in the study at the Chinese Sciences Academy University, Shenzhen Hospital in Guangdong Province from January 2012 to December 2016. All patients were diagnosed based on pathology.

In addition, 150 healthy subjects were selected randomly for the control group from the health screening program participants during the same period as the patients were enrolled. Individuals with a history of cancer or those related to the included patients were excluded. The controls and patients were frequency-matched for sex, age, and residential area.

The Institutional Ethics Committee of Chinese Science Academy University, Shenzhen Hospital approved this study. Informed consent was obtained from all the subjects included in this study before any study procedure.

### DNA extraction and genotyping

Genomic DNA was extracted from the NPC tissues and peripheral venous blood samples at the time of enrollment for genotyping using the QIAamp DNA FFPE Tissue Kit (Qiagen, Germany), according to the manufacturer’s instructions, at Ubiolab Genetics Technology Ltd. (Beijing, China).

Based on the DNA sequences (GenBank ID: 5967) of the human *REG1A* gene, six pairs of specific polymerase chain reaction (PCR) primers, covering the six exons, were designed using the Primer Premier v5.0 software to amplify the exons (Table 1).

**Table 1:** Primer sequences of PCR for the *reg1α* gene

Primer	Sequence (5′–3′)	Product length (bp)
REG1A-S1-F1	AAAGGCTCGTACTGGTGCC	637
REG1A-S1-R1	GAGACACCCACACCTTCAAATGTTTCTCTTGAGAGT	
REG1A-S1-F2	ATTTGAAGGTGTGGGTGTCTCAGAGGACCTTCCT	
REG1A-S1-R2	TGCTTGGGGATAGAGTGAAGTC	
REG1A-S2-F1	ACCCTGAGAGCCTCCTTTAATTG	661
REG1A-S2-R1	GATGCTGTCACTGACCACCAGGTTCTTTGTGCTG	
REG1A-S2-F2	TGGTGGTCAGTGACAGCATCATCACGGACATTACT	
REG1A-S2-R2	GAACCTCCTTCTTACTTCTCAAACC	
REG1A-S3-F1	TGAGTGACCACTGCCTCTGT	654
REG1A-S3-R1	TGGCTTTAGGACTCAGGACAAAAACCAAACAT	
REG1A-S3-F2	TGTCCTGAGTCCTAAAGCCAGGAGGGTCAT	
REG1A-S3-R2	CCAGGCATCAGCTGTGGAA	

S1-F1/R1 is used for the first exon amplification and S1-F2/R2 is used for the second exon. After that, both the products are taken as a template and F1/R2 is used as a primer for overlap amplification and the first and second exon sequences are obtained by product sequencing.

S2-F1/R1 is used for the third exon amplification and S2-F2/R2 is used for the fourth exon. After that, both the products are taken as a template, and F1/R2 is used as a primer to conduct overlap amplification and the third and fourth exon sequences are obtained by product sequencing.

S3-F1/R1 is used for the fifth exon amplification and S3-F2/R2 is used for the sixth exon. After that, both the products are taken as a template, and F1/R2 is used as a primer to conduct overlap amplification and the fifth and sixth exon sequences are obtained by product sequencing

*F* forward, *R* reverse, *S* slice

### Amplification and sequencing

The first PCR reaction was carried out with 2.5 μL of 10× buffer Gold (Applied Biosystems, USA), 200 μM each dNTP, 1.5 μM each primer, 1.25 U of AmpliTaq Gold Polymerase (Applied Biosystems, USA) and about 20 ng of DNA, in 25 μL total volume. Thermal cycling was performed in a Veriti system (Applied Biosystems, USA), and consisted of an initial 5 min denaturation step at 95 °C, 35 cycles of 30 s at 95 °C, 30 s at the 58 °C and 40 s extension step at 72 °C, followed by 5 min at 72 °C.

The second PCR was carried out with 2.5 μL of 10× buffer Gold (Applied Biosystems, USA), 200 μM each dNTP, 1.5 μM each primer, 1.25 U of AmpliTaq Gold Polymerase (Applied Biosystems, USA) and about 2 μL product of the first amplification, in 25 μL total volume. Thermal cycling was performed in a Veriti system (Applied Biosystems, USA), and consisted of an initial 5 min denaturation step at 95 °C, 35 cycles of 30 s at 95 °C, 30 s at the 58 °C and 40 s extension step at 72 °C, followed by 5 min at 72 °C.

The final PCR products were cleaned up using the QIAquick PCR purification kit (Qiagen, Germany) and then sequenced using the BigDye Terminator kit v3.1 (Applied Biosystems, USA) according to the manufacturer’s protocol. The *REG1A* SNPs Reg1A-14 (14C/T; exon 1), rs10165462 (20C/T; exon 1), rs117580393 (369G/T; exon 2), rs768985544 (1201A/G; exon 3), and rs12072 (2922T/C; exon 6) were examined.

### Statistical analysis

Genotype and minor allele frequency for the *REG1A* were counted, and the Hardy–Weinberg equilibrium was tested using the Chi-square test and the gPlink and Haploview software. The associations of *REG1A* polymorphisms with the risk of NPC were examined with univariable and multivariable logistic regression analyses (unconditional, or after adjustment for age and sex), and presented as odds ratio (OR) and 95% confidence interval (CI), using the SPSS software (version 19.0, IBM, Armonk, NY, USA). The minor allele frequency was calculated. Two-sided *p* values < 0.05 were considered statistically significant.

## Results

### Characteristics of NPC patients and controls

There were 172 male and 39 female patients, and the average age was 49.2 ± 5.8 years. There were 110 male and 40 female controls, and the average age was 48.3 ± 5.2. There were no significant differences between patients and controls with regard to age and sex (both *p* > 0.05).

DNA sequencing revealed the genotype variations in the exons 1, 2, 3 and 6 of *REG1A* gene (Table 2). The distribution of each *REG1A* gene polymorphism in total samples conformed to the Hardy–Weinberg equilibrium (*p* = 1 for REG1A-14, *p* = 1 for rs117580393, *p* = 1 for rs768985544 and *p* = 0.06995 for rs12072; supplementary table 1), except for re10165462 (*p* = 0.002; supplementary Table 1). In the genotype comparison using Chi-square test, only rs12072 showed a statistical difference between the cases and the controls (*p* = 0.0005) (Table 2), which was consistent with its different distributions in the case group (equilibrium) (*p* = 0.06995; supplementary table 1) versus the control group (*p* = 0.00066; supplementary Table 1).

**Table 2:** Distribution of polymorphisms in the *REG1A* gene and genotype analysis

rs number	Position	Position in *REG1A* allele 1/allele 2	Genotype^a^ comparision			Minor allele frequency (MAF)			Analysis^c^	
			Case (*n* = 211)	Control (*n* = 150)	*p*^b^	Case MAF (*n* = 211)	Control MAF (*n* = 150)	Total MAF	*p*	OR (95% CI)
Reg1A-14	Exon 1	14C/T	0/1/210	0/0/150	0.3989	0.002	0	0.0014	0.3988	NAN
rs10165462	Exon 1	20C/T	31/127/53	15/106/29	0.1167	0.448	0.453	0.45	0.8843	0.9782 (0.7265–1.317)
rs117580393	Exon 2	369G/T	0/4/207	0/0/150	0.09	0.009	0	0.0055	0.0908	NAN
rs768985544	Exon 3	1201A/G	0/1/210	0/1/149	0.8079	0.002	0.003	0.0028	0.8082	0.7102 (0.0443–11.3)
rs12072	Exon 6	2922T/C	33/117/61	35/52/63	0.0005	0.434	0.407	0.4224	0.4695	1.117 (0.8274–1.508)

*OR* odds ratio, *CI* confidence interval

^a^CC/CT/TT for Reg1A-14 and rs10165462; GG/GT/TT for rs117580393; AA/AG/GG for rs768985544; TT/TC/CC for rs12072

^b^Genotype was compared between the two groups using the Chi-square test

^c^Univariate logistic regression analysis for association of REG1A polymorphisms with risk of NPC

### Association of *REG1A* polymorphisms with risk of NPC

Table 3 presents the multivariable logistic regression analyses between the genotypes and the risk of NPC. The *REG1A* 14C/T, 20C/T, 20T/T, 369G/T, 1201A/G, and 2292C/C genotypes were not associated with the risk of NPC, either before or after adjustment for age and sex. On the other hand, the *REG1A* 2922C/T polymorphism was significantly associated with the risk of NPC [*p* = 0.003 (OR 0.406, 95% CI = 0.226–0.732)]. This association was still statistically significant after adjusting for age and sex [*p* = 0.003 (OR 0.419, 95% CI 0.235–0.746)] (Table 3).

**Table 3:** Multivariable logistic regression analysis for association of *REG1A* polymorphisms with the risk of NPC and adjustment for age and sex

Polymorphisms	*p*	OR	Exp(B) 95% CI		*p*_adjust_	OR	Exp(B) 95% CI	
			Lower	Upper			Lower	Upper
14C/T		9.070E−9	9.070E−9	9.070E−9		6.109E−9	6.109E−9	6.109E−9
20C/T	0.050	1.994	1.001	3.972	0.105	1.739	0.891	3.392
20T/T	0.535	1.284	0.582	2.835	0.789	1.110	0.517	2.383
369G/T	0.997	8.838E−16	0.000	.c		2.226E−9	2.226E−9	2.226E−9
1201A/G	0.998	50,540,608.419	0.000	.c	0.809	1.409	0.087	22.712
2292C/T	0.003	0.406	0.226	0.732	0.003	0.419	0.235	0.746
2292C/C	0.892	0.959	0.525	1.753	0.930	0.974	0.539	1.760

*OR* odds ratio, *CI* confidence interval

## Discussion

Genetic variations result in differences in gene function, which in turn lead to different susceptibility to diseases. Many studies have been conducted to investigate the genetic variants that contribute to NPC susceptibility, but the molecular mechanisms are not completely understood.

Genetic variations of the exons in the *REG1A* gene have been examined in small numbers of studies using a combination of restriction fragment length polymorphism (RFLP), single-strand conformation polymorphism (SSCP) and sequencing techniques, and no mutations were identified in the coding regions of *REG1Α* gene and to be associated with the development and progression of several types of pancreatic diseases [28, 29]. Those studies were restricted to a possible role of regulatory variants and some coding regions in the *REG1A* gene. Another study analyzed the coding region in 12 Thai patients with fibrocalculous pancreatic diabetes and 22 controls, but T-385C, a polymorphism in exon 1 (5′UTR) has been missed due to the inherent limitations of techniques like SSCP, and because not all coding region can be detected in some cases [29]

In the present study, to the best of our knowledge, we are the first to examine the *REG1A* variants in cancer by an extensive analysis of the six exons of *REG1A* using direct sequencing. Our observations revealed a statistically significant difference in allele frequency between these groups for 2922C/T, suggesting that an association exists between the allelic variations in *REG1A* and NPC susceptibility. Individuals with the 2922C/T genotype will have a higher risk for developing NPC than the ones who do not have it (OR 0.419, 95% CI 0.235–0.746).

Evidence suggests that genetic variants may enhance the promoter activity and can affect their biogenesis, processing, and target site binding in a variety of ways [30]. The mechanism of the 2922C/T in the development of NPC needs subsequent further exploration.

Although our study has shown that one polymorphism in the *REG1A* gene is associated with the development of NPC in a Cantonese-speaking population, some limitations should be considered. The sample size of the study was relatively small (211 patients and 150 controls), and perhaps resulted in rs10165462 not respecting the Hardy–Weinberg equilibrium. In addition, other factors associated with NPC (such as smoking, alcohol and Epstein–Barr virus infection) and the interactions with each other were not accounted for. Those factors would bias the observation. Therefore, large patient cohort studies are required to confirm our results.

## In summary

Our preliminary study provides the first evidence that the heterozygote 2922C/T polymorphism in the *REG1A* gene may be moderately associated with an increased risk for NPC. The identified *REG1A* rs12720T/C may be a predictive marker to identify patients with a high risk for NPC in a Southern Chinese population. The exact mechanism that polymorphism of *REG1A* enhances the risk of NPC requires further investigation.

## Electronic supplementary material

Below is the link to the electronic supplementary material.

Supplementary file1 (DOCX 14 kb)

## References

[1] Siegel RL, Miller KD, Jemal A (2016). Cancer statistics, 2016. CA Cancer J Clin.

[2] Yoshizaki T, Ito M, Murono S, Wakisaka N, Kondo S, Endo K (2012). Current understanding and management of nasopharyngeal carcinoma. Auris Nasus Larynx.

[3] Liu Z, Fang F, Chang ET, Ye W (2015). Cancer risk in the relatives of patients with nasopharyngeal carcinoma—a register-based cohort study in Sweden. Br J Cancer.

[4] Chen CJ, Liang KY, Chang YS, Wang YF, Hsieh T, Hsu MM, Chen JY, Liu MY (1990). Multiple risk factors of nasopharyngeal carcinoma: Epstein–Barr virus, malarial infection, cigarette smoking and familial tendency. Anticancer Res.

[5] Schottenfeld D, Fraumeni JF (2006). Cancer epidemiology and prevention.

[6] Zeng L, Luo S, Li X, Lu M, Li H, Li T, Wang G, Lyu X, Jia W, Dong Z, Jiang Q, Shen Z, Huang GL, He Z (2017). Functional PIN1 promoter polymorphisms associated with risk of nasopharyngeal carcinoma in Southern Chinese populations. Sci Rep.

[7] Yang Y, Liu W, Zhao Z, Zhang Y, Xiao H, Luo B (2017). Filaggrin gene polymorphism associated with Epstein–Barr virus-associated tumors in China. Virus Genes.

[8] Deng L, Zhao XR, Pan KF, Wang Y, Deng XY, Lü YY, Cao Y (2002). Cyclin D1 polymorphism and the susceptibility to NPC using DHPLC. Sheng Wu Hua Xue Yu Sheng Wu Wu Li Xue Bao (Shanghai).

[9] Cho EY, Hildesheim A, Chen CJ, Hsu MM, Chen IH, Mittl BF, Levine PH, Liu MY, Chen JY, Brinton LA, Cheng YJ, Yang CS (2003). Nasopharyngeal carcinoma and genetic polymorphisms of DNA repair enzymes XRCC1and hOGG1. Cancer Epidemiol Biomark Prev.

[10] Guo XB, Ma WL, Liu LJ, Huang YL, Wang J, Huang LH, Peng XD, Yin JY, Li JG, Chen SJ, Yang GP, Wang H, Guo CX (2017). Effects of gene polymorphisms in the endoplasmic reticulum stress pathway on clinical outcomes of chemoradiotherapy in Chinese patients with nasopharyngeal carcinoma. Acta Pharmacol Sin.

[11] Guo C, Huang Y, Yu J, Liu L, Gong X, Huang M, Jiang C, Liao Y, Huang L, Yang G, Li J (2017). The impacts of single nucleotide polymorphisms in genes of cell cycle and NF-kB pathways on the efficacy and acute toxicities of radiotherapy in patients with nasopharyngeal carcinoma. Oncotarget.

[12] Ma WL, Liu R, Huang LH, Zou C, Huang J, Wang J, Chen SJ, Meng XG, Yang JK, Li H, Yang GP, Guo CX (2017). Impact of polymorphisms in angiogenesis-related genes on clinical outcomes of radiotherapy in patients with nasopharyngeal carcinoma. Clin Exp Pharmacol Physiol.

[13] Tan J, Jiang L, Cheng X, Wang C, Chen J, Huang X, Xie P, Xia D, Wang R, Zhang Y (2017). Association between VEGF-460T/C gene polymorphism and clinical outcomes of nasopharyngeal carcinoma treated with intensity-modulated radiation therapy. Onco Targets Ther.

[14] Tse KP, Tsang NM, Chen KD, Li HP, Liang Y, Hsueh C, Chang KP, Yu JS, Hao SP, Hsieh LL, Chang YS (2007). MCP-1 promoter polymorphism at 2518 is associated with metastasis of nasopharyngeal carcinoma after treatment. Clin Cancer Res.

[15] Ghandri N, Gabbouj S, Farhat K, Bouaouina N, Abdelaziz H, Nouri A, Chouchane L, Hassen E (2011). Association of HLA-G polymorphisms with nasopharyngeal carcinoma risk and clinical outcome. Hum Immunol.

[16] Zhang YW, Ding LS, Lai MD (2003). Reg gene family and human diseases. World J Gastroenterol.

[17] Carrere J, Guy-Crotte O, Gaia E, Figarella C (1999). Immunoreactive pancreatic Reg protein insera from cystic fibrosis patients with and without pancreatic in sufficiency. Gut.

[18] Fukui H, Fujii S, Takeda J, Kayahara T, Sekikawa A, Nanakin A, Suzuki K, Hisatsune H, Seno H, Sawada M, Fujimori T, Chiba T (2004). Expression of RegIa protein in human gastric cancers. Digestion.

[19] Rechreche H, Montalto G, Mallo GV, Vasseur S, Marasa L, Soubeyran P, Dagorn JC, Iovanna JL (1999). pap, regIa and regIb mRNAs are concomitantly up-regulated during human colorectal carcinogenesis. Int J Cancer.

[20] Zhou L, Zhang R, Wang L, Shen S, Okamoto H, Sugawara A, Xia L, Wang X, Noguchi N, Yoshikawa T, Uruno A, Yao W, Yuan Y (2010). Upregulation of REG I alpha accelerates tumor progression in pancreatic cancer with diabetes. Int J Cancer.

[21] Sasaki Y, Minamiya Y, Takahashi N, Nakagawa T, Katayose Y, Ito A, Saito H, Motoyama S, Ogawa J (2008). REG1A expression is an independent factor predictive of poor prognosis in patients with breast cancer. Ann Surg Oncol.

[22] Minamiya Y, Kawai H, Saito H, Ito M, Hosono Y, Motoyama S, Katayose Y, Takahashi N, Ogawa J (2008). REG1A expression is an independent factor predictive of poor prognosis in patients with non-small cell lung cancer. Lung Cancer.

[23] Geng J, Fan J, Wang P, Fang ZJ, Xia GW, Jiang HW, Chen G, Ding Q (2009). REG1A predicts recurrence in stage Ta/T1 bladder cancer. Eur J Surg Oncol.

[24] Yuan RH, Jeng YM, Chen HL, Hsieh FJ, Yang CY, Lee PH, Hsu HC (2005). Opposite roles of human pancreatitis-associated protein and REG1A expression in hepatocellular carcinoma: association of pancreatitis-associated protein expression with low-stage hepatocellular carcinoma, beta-catenin mutation, and favorable prognosis. Clin Cancer Res.

[25] Aboshanif M, Kawasaki Y, Omori Y, Suzuki S, Honda K, Motoyama S, Ishikawa K (2016). Prognostic role of regenerating gene-I in patients with stage-IV head and neck squamous cell carcinoma. Diagn Pathol.

[26] Hayashi K, Motoyama S, Sugiyama T, Izumi J, Anbai A, Nanjo H, Watanabe H, Maruyama K, Minamiya Y, Koyota S, Koizumi Y, Takasawa S, Murata K, Ogawa J (2008). REGIalpha is a reliable marker of chemoradiosensitivity in squamous cell esophageal cancer patients. Ann Surg Oncol.

[27] Xing H, Chen X, Han Y (2017). Role of regenerating gene IA expression on local invasion and survival in nasopharyngeal carcinoma. Biol Res.

[28] Hawrami K, Mohan V, Bone A, Hitman GA (1997). Analysis of islet regenerating (reg) gene polymorphisms in fibrocalculous pancreatic diabetes. Pancreas.

[29] Mahurkar S, Bhaskar S, Reddy DN, Rao GV, Chandak GR (2007). Comprehensive screening for reg1alpha gene rules out association with tropical calcific pancreatitis. World J Gastroenterol.

[30] Ryan BM, Robles AI, Harris CC (2010). Genetic variation in microRNA net-works: the implications for cancer research. Nat Rev Cancer.

