# Supplementary material for: Association of regenerating gene 1A single-nucleotide polymorphisms and nasopharyngeal carcinoma susceptibility in southern Chinese population
Source: Eur Arch Otorhinolaryngol. 2019 Sep 20;277(1):221–6. doi: 10.1007/s00405-019-05645-9 (PMC6942584; doi:10.1007/s00405-019-05645-9)
Supplement: Supplementary file 1 — Supplementary file1 (DOCX 14 kb) [file 405_2019_5645_MOESM1_ESM.docx]

| SNP | TEST | GENO | O(HET) | E(HET) | P_HWD |
| --- | --- | --- | --- | --- | --- |
| REG1A-14 | ALL | 0/1/360 | 0.00277 | 0.002766 | 1 |
| REG1A-14 | AFF | 0/1/210 | 0.004739 | 0.004728 | 1 |
| REG1A-14 | UNAFF | 0/0/150 | 0 | 0 | 1 |
| rs10165462 | ALL | 46/233/82 | 0.6454 | 0.495 | 7.94E-09 |
| rs10165462 | AFF | 31/127/53 | 0.6019 | 0.4946 | 0.002159 |
| rs10165462 | UNAFF | 15/106/29 | 0.7067 | 0.4956 | 2.24E-07 |
| rs117580393 | ALL | 0/4/357 | 0.01108 | 0.01102 | 1 |
| rs117580393 | AFF | 0/4/207 | 0.01896 | 0.01878 | 1 |
| rs117580393 | UNAFF | 0/0/150 | 0 | 0 | 1 |
| rs768985544 | ALL | 0/2/359 | 0.00554 | 0.005525 | 1 |
| rs768985544 | AFF | 0/1/210 | 0.004739 | 0.004728 | 1 |
| rs768985544 | UNAFF | 0/1/149 | 0.006667 | 0.006644 | 1 |
| rs12072 | ALL | 68/169/124 | 0.4681 | 0.488 | 0.4508 |
| rs12072 | AFF | 33/117/61 | 0.5545 | 0.4912 | 0.06995 |
| rs12072 | UNAFF | 35/52/63 | 0.3467 | 0.4826 | 0.00066 |

Supplymentary table 1. Hardy-Weinberg equilibrium Analysis
